# An Abnormal Presentation of Rocky Mountain Spotted Fever: A Case Report

**DOI:** 10.7759/cureus.57319

**Published:** 2024-03-31

**Authors:** Joshua J Nelson, Kaedon Buchmiller, Michael J Valentine, Kirthika Lakshmanan, Ankur Kayastha, Jagjot S Dhingra, Riley G Fisher, Connor A Parry, Annie K Konrad, Arman Mughal, Carol E Kirila

**Affiliations:** 1 College of Osteopathic Medicine, Kansas City University, Kansas City, USA; 2 Medicine, Kansas City University, Kansas City, USA; 3 General Practice, Brigham Young University, Provo, USA; 4 Primary Care/Internal Medicine, Kansas City University, Kansas City, USA

**Keywords:** unusual presentation, confusion, altered mental state, hypertension, tick bite, rocky mountain spotted fever

## Abstract

The intracellular coccobacilli *Rickettsia rickettsii* causes Rocky Mountain Spotted Fever, a potentially fatal illness. This bacterium is transmitted to humans through a tick vector. Patients classically present with a triad of symptoms, including fever, headache, and a rash that begins on the extremities and spreads proximally to the trunk. Diagnosis of this disease can prove difficult when patients have unusual symptoms, such as hypertensive crisis. In this case report, we present a 29-year-old male who arrived at the emergency room with altered mental status and a hypertensive crisis after his family reported one week of changes in his behavior. The patient had no evidence of ticks, tick bites, fever, or rash. Positive findings in the emergency room included a WBC of 14.9 × 109. All other physical exams, imaging, and laboratory findings were non-contributory. The patient was promptly given IV hydralazine to control his blood pressure and empiric IV ceftriaxone for potential infection, and he was admitted for observation. Over the course of three days, WBC levels decreased, and his altered mental status improved. On day 3, the patient remembered a tick crawling across his hand, and this prompted the ordering of immunoglobulin levels for tick-borne illnesses. IgM for RMSF was positive. This case presentation illustrates the need for clinicians to keep the potential diagnosis of RMSF high on the differential, even in the presence of a paucity of symptoms, as prompt treatment with doxycycline can be lifesaving. This case may also be one of the first reported in the literature of hypertension being a symptom of Rocky Mountain Spotted Fever. It is plausible, however, that this patient’s hypertension was due to an acute stress response.

## Introduction

Rocky Mountain Spotted Fever (RMSF) is a potentially fatal illness caused by the Gram-negative intracellular coccobacilli *Rickettsia rickettsii*. This bacterium is transmitted to humans through a tick vector. In the United States, the American dog tick (*Dermacentor variabilis*), the Rocky Mountain wood tick (*Dermacentor andersoni*), and the Brown dog tick (Rhipicephalus sanguineus) are among some of the common ticks that transmit the bacteria [1]. Patients inoculated with the disease classically present with a triad of symptoms that include fever, headache, and a rash that begins on the extremities and spreads proximally to involve the trunk [2]. This triad of symptoms, however, has been found to represent only up to 18% of patients on their initial visit to a clinician [3]. Many patients experience a myriad of other symptoms that may complicate the diagnosis. These symptoms include, but are not limited to, decreased appetite, chills, sore throat, confusion, abdominal pain, nausea, vomiting, body aches, sensitivity to light, and hypotension. Additionally, a history of tick bite or exposure is only elicited in 50% to 60% of patients [4], which could cause clinicians to prematurely rule out RMSF. Abnormal laboratory findings such as hyponatremia, lymphopenia, thrombocytopenia, and coagulopathy may also be detected later in the disease process [5]. Immunoglobulin titers can be ordered as a confirmatory study. With mortality rates of untreated patients presenting with RMSF being as high as 20% to 30% without prompt antibiotic treatment [1], it is critical to keep this disease high on the differential diagnosis when clinical suspicion is warranted. In this case report, we present a previously healthy patient with hypertensive encephalopathy secondary to RMSF.

## Case presentation

A 29-year-old male presented to the emergency department with altered mental status and a hypertensive crisis, with an upper limit blood pressure recording at 218/80 mmHg. Concerns were raised by his family regarding recent changes in behavior over the past week, as the patient often repeated himself, stating, "I just don't feel like myself." The patient is employed as a mailman in a rural area with exposure to tall, weed-like grass and low, overhanging tree branches. Upon questioning, he did not recall any insect bites, skin rash, or subjective fever. No ticks or tick bites were observed. Common symptoms of meningitis or encephalitis, including neck stiffness, photosensitivity, and noise sensitivity, were absent. The neurological examination was unremarkable, and a neurology consultation yielded no significant findings. Head CT, head and neck CTA, CXR, brain MRI, urinalysis, and acute meningitis panel were noncontributory. Positive laboratory findings included a leukocytosis with a WBC count of 14.9 × 10^9^/L (normal WBC reference range: 4.5 × 10^9^/L to 10.1 × 10^9^/L), and cerebrospinal fluid (CSF) revealed 102 red blood cells in a colorless fluid. Due to the elevated blood pressure, the patient was promptly given hydralazine to mitigate cardiovascular risk and end-organ damage. The differential diagnosis consisted of metabolic encephalopathy, sepsis, hypoxic conditions, and infectious etiology. Given the acuity of the patient's symptoms in the context of his occupation and environmental exposure, an infectious etiology was suspected. Empirical antibiotic therapy consisting of 1 g/50 ml of intravenous (IV) ceftriaxone was begun based on infectious disease recommendations. Subsequently, the patient was admitted for further observation and management.

During hospitalization, serial monitoring of leukocyte counts revealed fluctuating values coinciding with gradual improvement. His initial WBC count decreased to 12.2 × 10^9^/L, increased to 12.4 × 10^9^/L, and then decreased to 10.7 × 10^9^/L on days 1, 2, and 3, respectively. His altered mental status continually improved each day.

**Figure 1:**
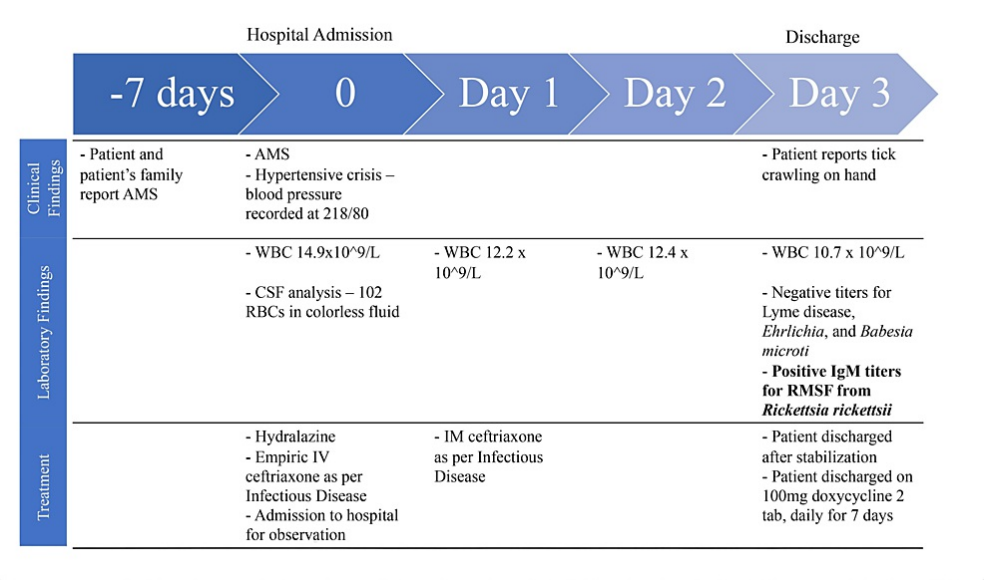
Hospital course timeline including clinical findings, laboratory findings, and treatment. The timeline begins seven days prior to admission and the three day hospital stay. AMS: altered mental status; WBC: white blood cells (normal WBC reference range: 4.5 x 10^9^/L to 10.1 x 10^9^/L); CSF: cerebrospinal fluid; IV: intravenous; IM: intramuscular; IgM: immunoglobulin M; RMSF: Rocky Mountain Spotted Fever.

Infectious disease was consulted again on day 1 of observation and recommended administering an extra intramuscular injection of ceftriaxone. On the third day of observation, the patient reported a tick crawling over his left hand. With this new information, titers for tick-borne illnesses were ordered. Titers for Lyme disease, Ehrlichia, and Babesia microti returned negative. However, IgM titers for RMSF from Rickettsia rickettsii were positive. After patient stabilization and the return of WBCs to normal range, the patient was discharged with instructions to take two 100 mg doxycycline tablets per day for seven days and to follow up with his primary care provider. A hospital course timeline is found in Figure 1.

## Discussion

Rocky Mountain Spotted Fever is regarded as the most lethal rickettsial disease in the current literature [6]. The disease was first acknowledged in the early 1800s [7]. The incidence of nationally reported cases has been rising from a low in 1998 at 1.3 cases per million to a high of 19.3 cases per million in 2017 [8]. Cases are reported throughout each month of the year; however, the majority occur from May through August [9]. Notably, while the Rocky Mountains are geographically located in the Western portion of the United States, cases of RMSF are more likely to occur in the Midwest and Eastern regions. Moreover, geographical location should not be used as a means for disease exclusion since more than 60% of cases arise in North Carolina, Oklahoma, Arkansas, Tennessee, and Missouri [9]. Of the tick species that carry the disease, all have different geographic distributions throughout the United States. The Rocky Mountain wood tick (*Dermacentor andersoni*) can be found throughout the Rocky Mountain region. The American dog tick (*Dermacentor variabilis*) can be found east of the Rocky Mountains and on portions of the Pacific coast. The brown dog tick (*Rhipicephalus sanguineus*) can be found throughout the U.S. and serves as the primary vector for RMSF in the southwestern United States [9].

As mentioned previously, *Rickettsia rickettsii* is a potentially fatal infection that occurs rapidly after the tick bite and inoculation of the human host. The Gram-negative, obligate intracellular coccobacilli enter the bloodstream and replicate inside vascular endothelial cells lining the small and medium vessels of the body. Damage to these cells subsequently causes a vasculitis-like disease, leading to systemic inflammation, loss of barrier function, and altered vascular permeability throughout the body [1]. This process manifests clinically about 2 to 14 days after infection with symptoms such as fever, myalgia, nausea, vomiting, anorexia, abdominal pain, headache, confusion, low blood pressure, and a petechial rash. The typical rash associated with RMSF usually appears from days 3 to 5, begins on the wrists and ankles, and forms small pink macules, which then spread centrally and turn into darker papules. In one study, 261 out of 322 patients (80%) were found to have a rash from RMSF [10]. Notably, our patient did not present with the typical rash. The only symptoms our patient had were altered mental status, elevated blood pressure, and leukocytosis. Additionally, if RMSF is left untreated, patients can accumulate organ and tissue damage that may ultimately prove fatal. Death is likely to be avoided if a diagnosis is made promptly. 

Due to the wide variety of nonspecific symptoms, the diagnosis of Rocky Mountain Spotted Fever can be challenging. Diagnosis is usually made clinically via immunoglobulin M (IgM) and/or immunoglobulin G (IgG) titers [1]. Clinical suspicion of RMSF should remain high despite normal IgM and IgG levels if evaluated early in the disease course [1]. Other tests that can be used in the diagnosis of RMSF are polymerase chain reaction (PCR), skin biopsy immunostaining, and microbial culture techniques. These tests are less often utilized due to limited availability [4]. Lab values such as thrombocytopenia, leukocytosis, and hyponatremia are also of limited use since they can be negative on presentation. The first-line treatment for RMSF in all age groups is doxycycline [11], and treatment should not be delayed if there is any clinical suspicion of the disease. In this case, the patient was given empiric ceftriaxone pending the results of further labs. Upon discovery of RMSF, the patient was then given a seven-day course of oral doxycycline according to the guidelines established by the Centers for Disease Control and Prevention [12].

The case presented provides a peculiar presentation of RMSF since the patient only displayed symptoms of hypertension, confusion, and an elevated white blood count with no history of tick bite, fever, or rash. In fact, hypertension is most unusual because the damage caused by RMSF to vascular endothelial cells usually causes leaky blood vessels, potentially resulting in sepsis and subsequent hypotension. A theoretical cause of hypertension in this patient may be a physiologic stress response caused by the infection. To the best of our knowledge, there have been no cases reported in the literature of RMSF causing hypertension; however, this does not rule out the possibility of it being a symptom of the disease. The patient's leukocytosis was a significant clinical clue that there was an infectious process occurring. This prompted the rapid administration of ceftriaxone, pending lab results. The low threshold for starting empiric antibiotics likely positively impacted the patient from further complications of RMSF. Clinicians are recommended to maintain a reasonably low clinical threshold for antibiotic administration in the setting of unknown etiology but present leukocytosis. Additionally, in the setting of absent tick bites, clinicians are recommended to keep RMSF within the differential diagnosis [13].

## Conclusions

Given the potential lethal outcomes associated with RMSF, prompt action is critical for patients who have been exposed. Healthcare providers must remain vigilant regarding this tick-borne disease, particularly during the periods of spring and summer. It is important to recognize that a documented history of a tick bite may not always accompany this tick-borne illness. Recent travel may be helpful in discerning geographical associations; however, geographical location should not be used as a means of disease exclusion. Doxycycline continues to be the mainstay of antibiotic therapy for treatment. This case illustrates the necessity for clinicians to maintain a low threshold of suspicion for this disease as a potential cause of altered mental status, hypertension, and leukocytosis. Additionally, this case demonstrates the difficulty of diagnosing RMSF from a paucity of clinical symptoms, laboratory results, and imaging findings. The severe hypertension displayed in this patient may be a rare symptom of Rocky Mountain Spotted Fever; however, more evidence is needed to explore this possible association.
